# Supplementary figures and images for: The ubiquitin-conjugating enzyme UBE2D maintains a youthful proteome and ensures protein quality control during aging by sustaining proteasome activity
Source: PLoS Biol. 2025 Jan 29;23(1):e3002998. doi: 10.1371/journal.pbio.3002998 (PMC11778781; doi:10.1371/journal.pbio.3002998)

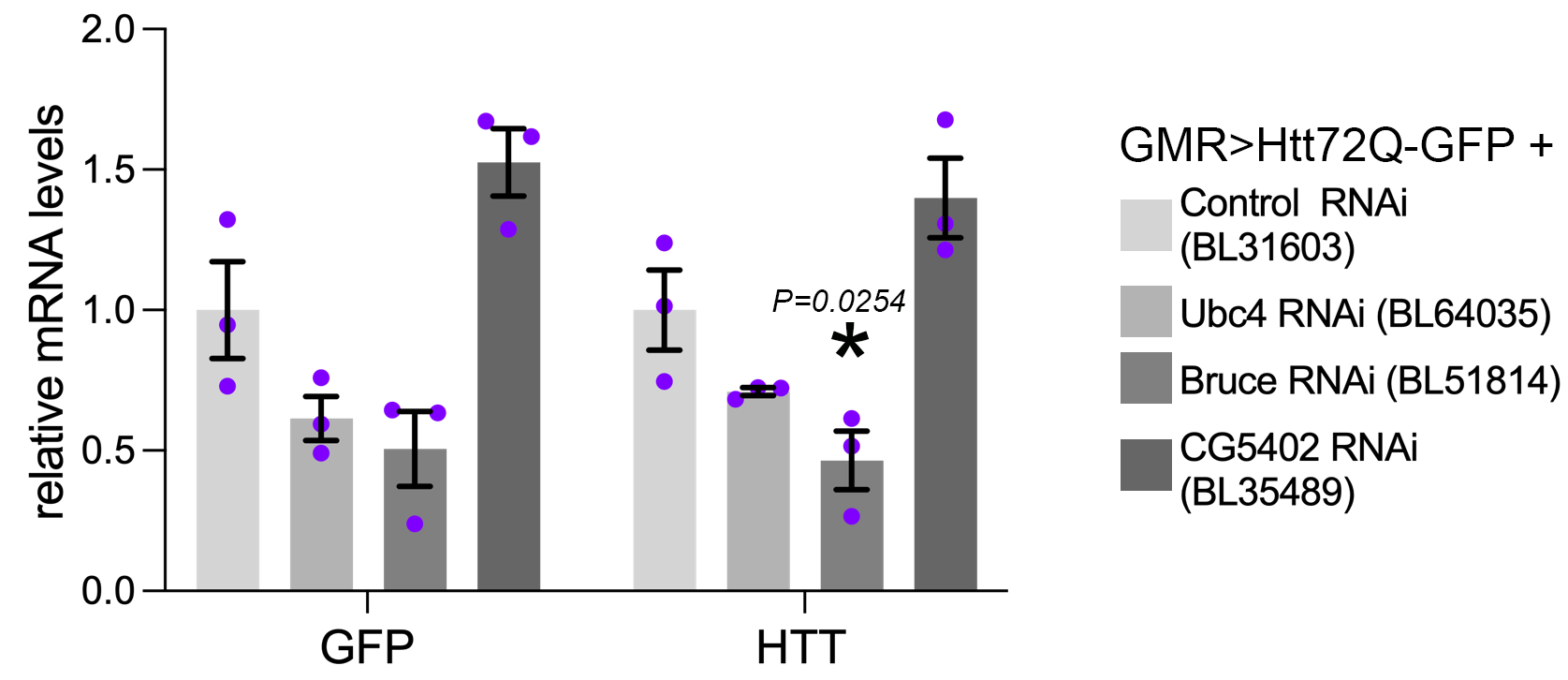

Supplement: S1 Fig — qRT-PCR from the heads of flies that express huntingtin-polyQ72-GFP in the retina (with GMR-Gal4) alongside a control or RNAi for some of the E2s that scored in the Htt screen (Fig 1): Ubc4, Bruce, and CG5402. This gene expression analysis indicates that there is a substantial decline in the transgenic expression of huntingtin-polyQ72-GFP (as indicated by the GFP and Htt mRNA levels) upon knockdown of Ubc4 and Bruce, whereas there is a partial increase in response to CG5402 RNAi. The graphs display the mean ± SEM with n = 3 (biological replicates). Statistical analysis was done with one-way ANOVA and Dunnett’s multiple comparisons test; *p < 0.05. These findings indicate that the knockdown of Ubc4, Bruce, and CG5402 may modulate the amount of GFP-tagged huntingtin-polyQ aggregates via changes in the expression of the Htt-polyQ72-GFP transgene: on this basis, these E2s were not considered for further analyses. Mechanistically, these E2s may modulate transgenic expression by regulating histone ubiquitination and degradation (which generally alters transcriptional activity), as previously shown for the Ubc4 ortholog UBE2K (PMID: 32451438). Related to Fig 1. The data underlying the graphs shown in this figure can be found in the S6 Data file. (TIFF) [file pbio.3002998.s001.tiff]

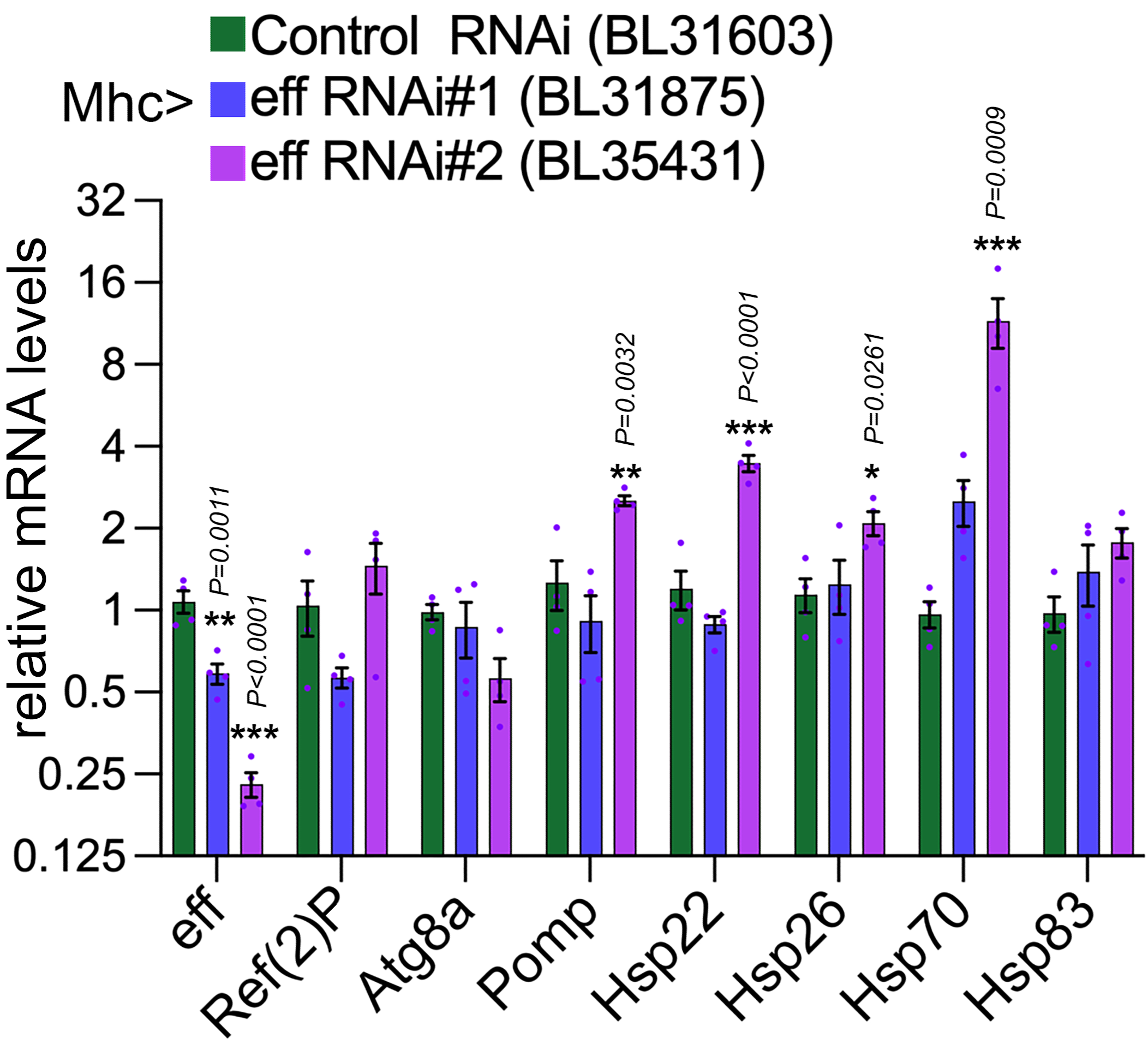

Supplement: S2 Fig — qRT-PCR analysis of skeletal muscle with effRNAi and controlRNAi. Knockdown of eff up-regulates the expression of the proteasome assembly factor Pomp and of the chaperones Hsp22, Hsp26, and Hsp70. The graphs display the mean ± SEM with n = 3 (biological replicates). Statistical analysis was done with one-way ANOVA and Dunnett’s multiple comparisons test; *p < 0.05; **p < 0.01; ***p < 0.001. These findings suggest that a stress response (presumably related to the unfolded protein response or the proteasome stress response) is triggered by the decline in protein quality control due to UBE2D/eff knockdown. Interestingly, this response is primarily induced only by the stronger effRNAi line (BL#35431), suggesting that there could be a threshold of eff knockdown that is required for the transcriptional induction of this response. The data underlying the graphs shown in this figure can be found in the S6 Data file. (TIF) [file pbio.3002998.s002.tif]
