# Supplementary material for: The ubiquitin-conjugating enzyme UBE2D maintains a youthful proteome and ensures protein quality control during aging by sustaining proteasome activity
Source: PLoS Biol. 2025 Jan 29;23(1):e3002998. doi: 10.1371/journal.pbio.3002998 (PMC11778781; doi:10.1371/journal.pbio.3002998)
Supplement: S1 Raw Images — (PDF) [file pbio.3002998.s009.pdf]

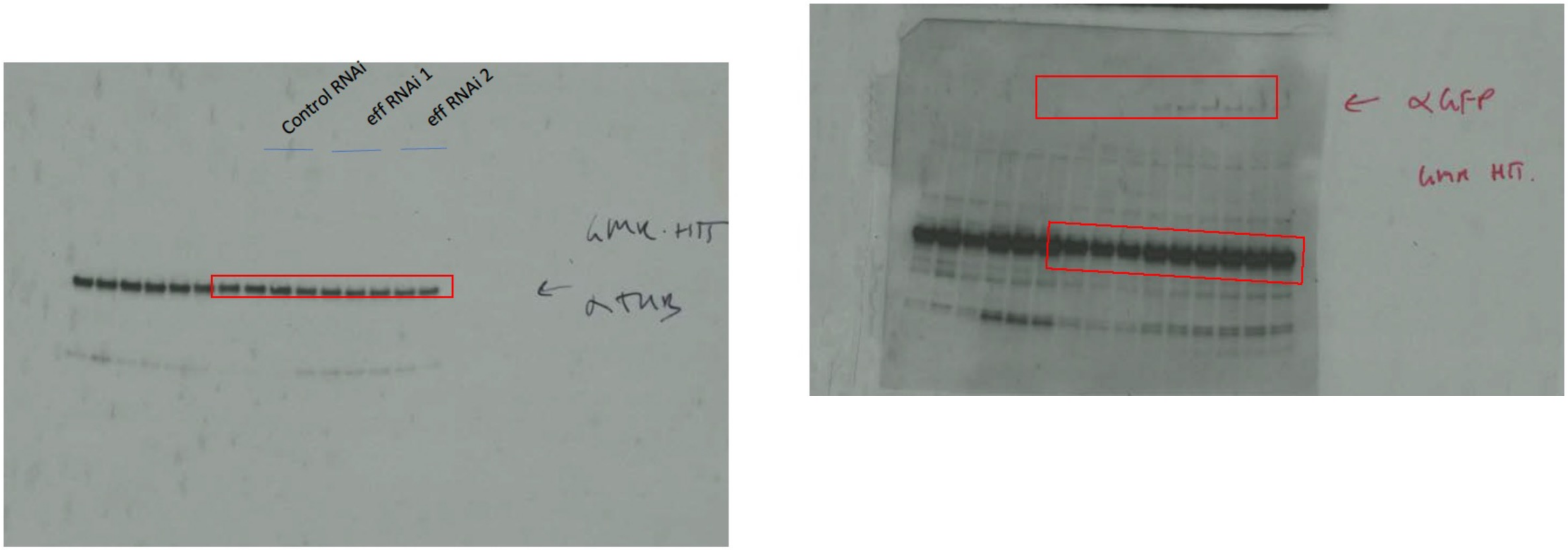

Fig. 1E

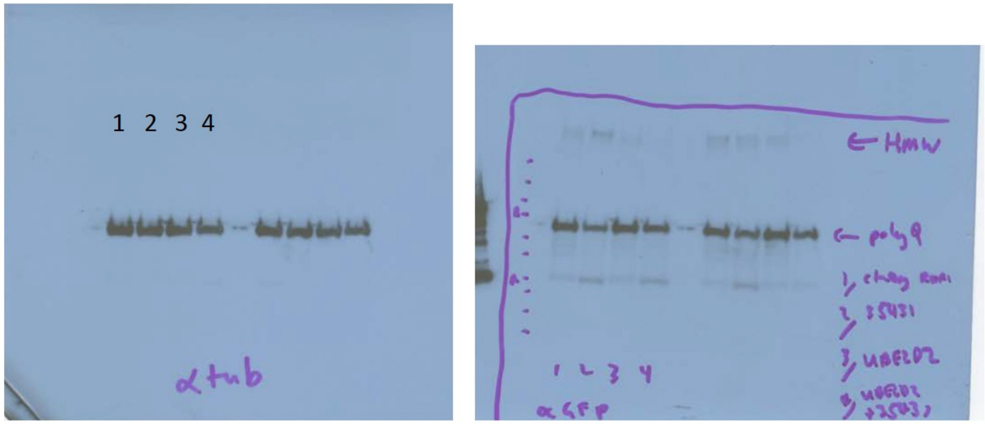

Figure 2C

Fig. 2C

detergent-soluble fractions

ubiquitin

Ref(2)P

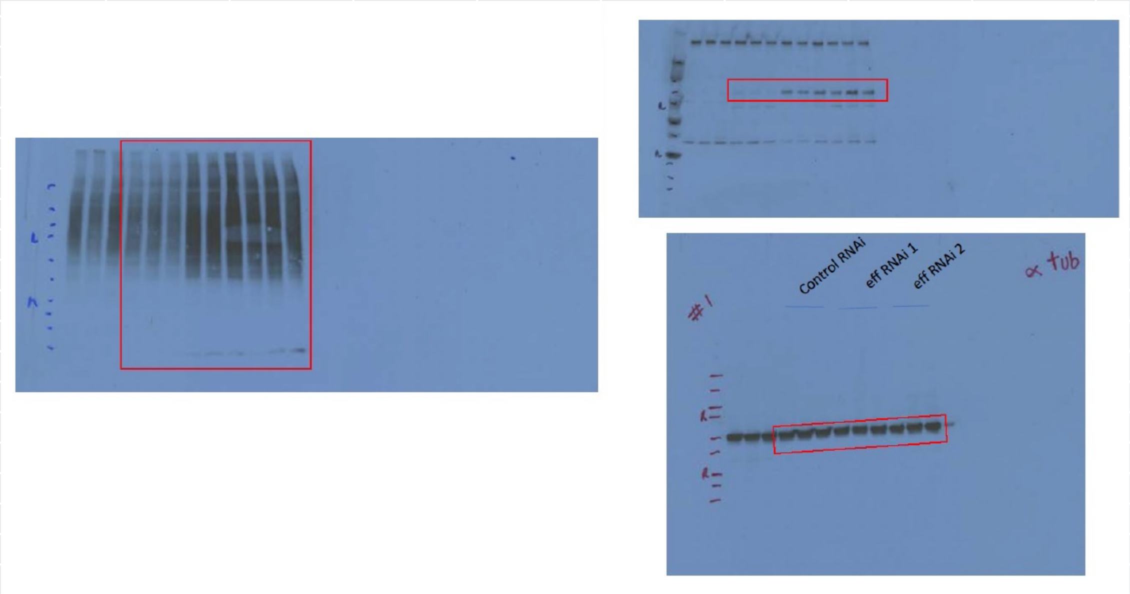

tubulin

Fig. 3E – part 1

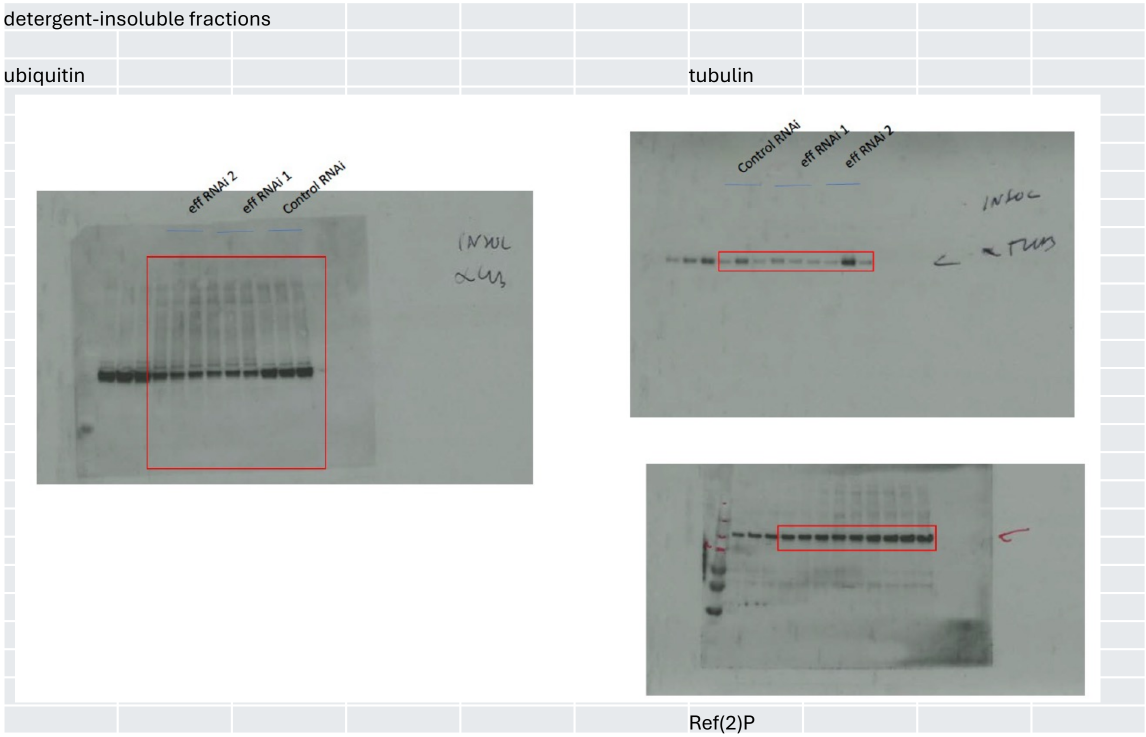
detergent-insoluble fractions
ubiquitin
tubulin
eff RNAi 2
eff RNAi 1
Control RNAi
Control RNAi
eff RNAi 1
eff RNAi 2
Ref(2)P

Fig. 3E – part 2

detergent-soluble fractions

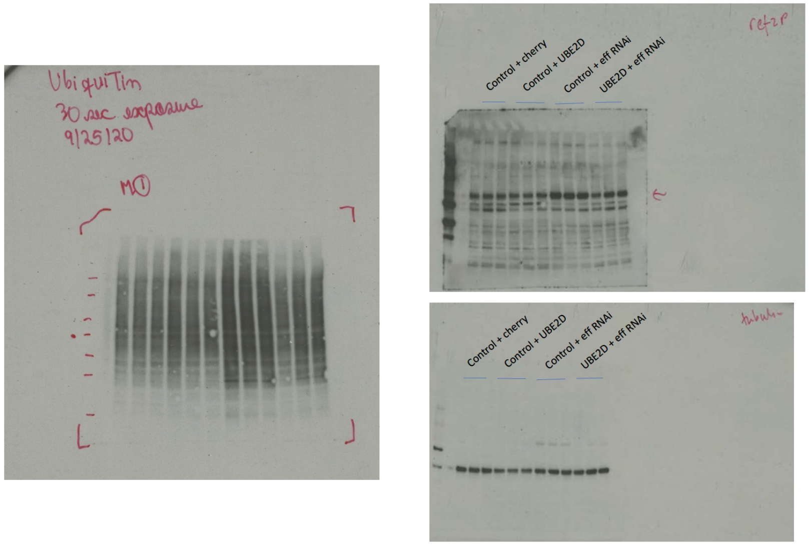

Figure 4A
Soluble

Fig. 4A – part 1

detergent-insoluble fractions

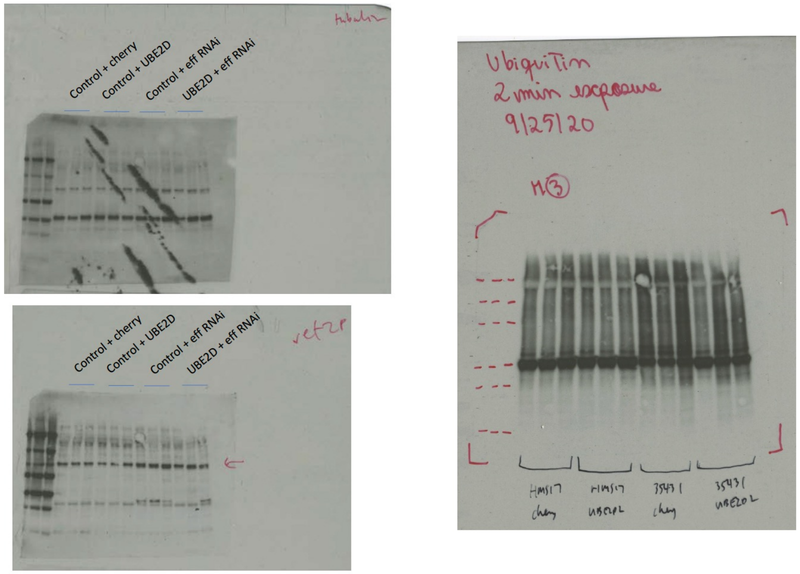

Figure 4A
Insoluble

Fig. 4A – part 2

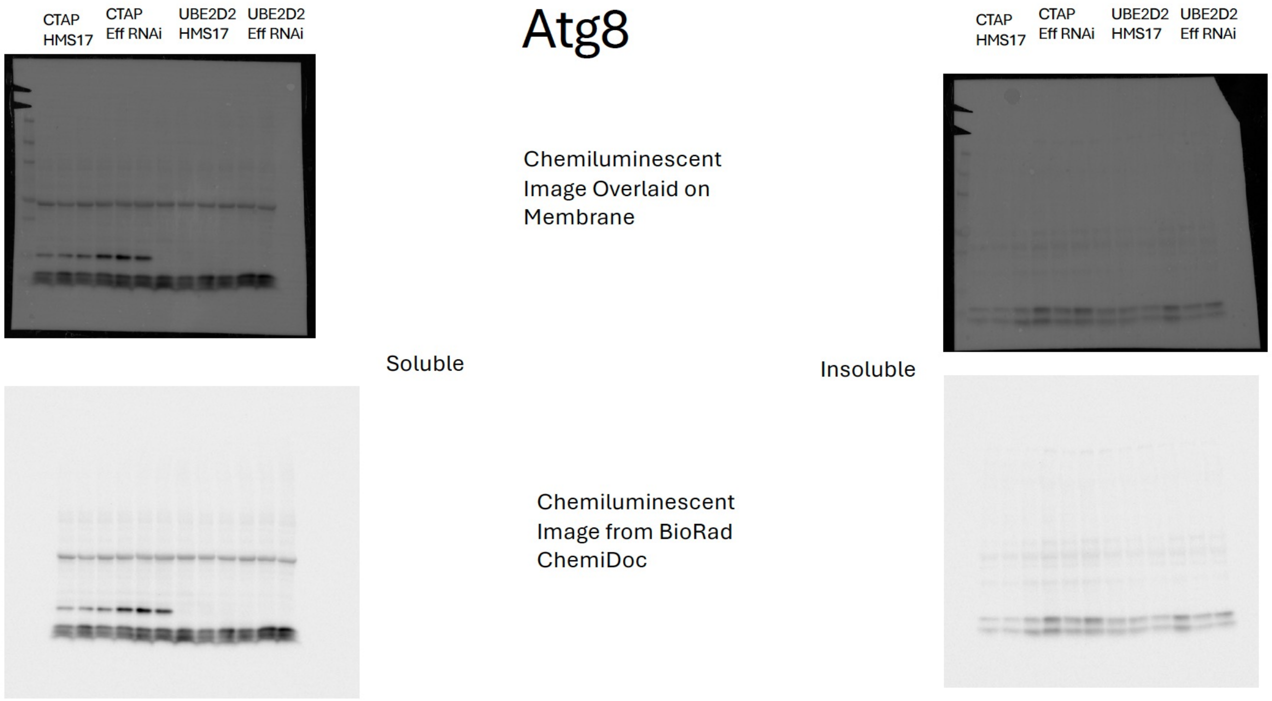

Fig. 5D

Fig. 5E

part 1

Alpha Tubulin

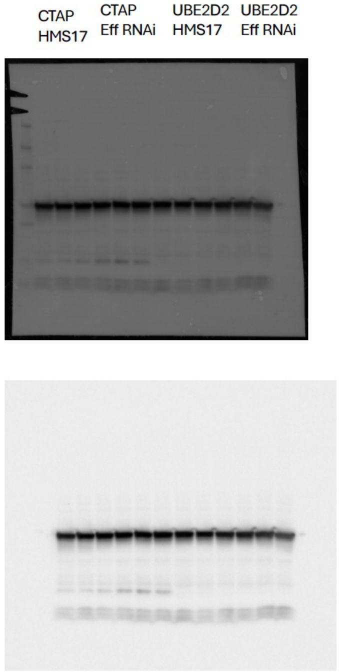

Chemiluminescent Image Overlaid on Membrane

Soluble

Chemiluminescent Image from BioRad ChemiDoc

Fig. 5D

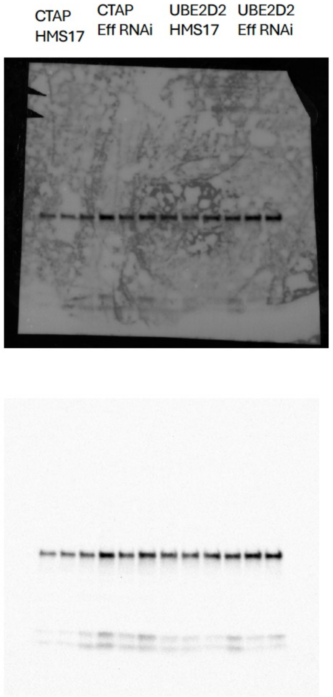

Insoluble

Fig. 5E

part 2

Ponceau

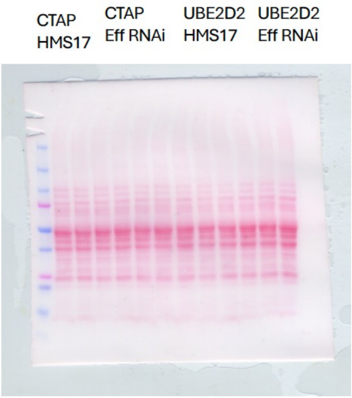

Soluble

Fig. 5D

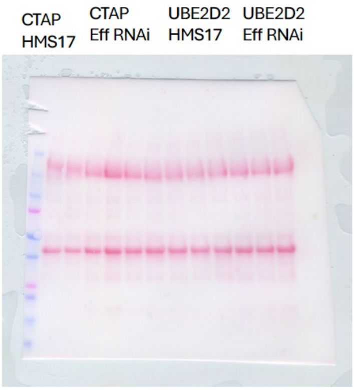

Insoluble

Fig. 5E

part 3
