# Supplementary material for: The ubiquitin-conjugating enzyme UBE2D maintains a youthful proteome and ensures protein quality control during aging by sustaining proteasome activity
Source: PLoS Biol. 2025 Jan 29;23(1):e3002998. doi: 10.1371/journal.pbio.3002998 (PMC11778781; doi:10.1371/journal.pbio.3002998)
Supplement: Attachment — Submitted filename: Review PBIOLOGY-D-24-01321R1.pdf [file pbio.3002998.s010.pdf]

PLOS Biology review

Manuscript: PBIOLOGY-D-24-01321R1

Research Article: The ubiquitin-conjugating enzyme UBE2D/eff maintains a youthful proteome and ensures protein quality control during aging.

Fabio Demontis, PhD.

Overall Recommendation: Minor Revisions. This is an interesting paper with good experimental data. Minor experimental and a few editorial additions will make it suitable for publication in PLOS Biology.

Novelty: This is an exciting line of investigation, and the hypothesis and experimental plan is novel.

Significance is Moderate. The significance of this work could be increased if it can be shown where eff E2 enzyme is functions, and whether it has a general effect of overall aggregate turnover, or more specifically on proteasome function, or some other key step in protein turnover.

Technical merits: The experimental approach is sound, and the results are convincing. However, some improvements are recommended. Measurement of proteasome activity will determine if eff affects the function of this multicatalytic protease. Measurement of proteasome assembly will similarly test this. These experiments are important for completion of this solid body of work, and can be performed simultaneously in a native gel. The authors should explain why they selected eff for further investigation, rather than Ubc6, which seemed to have a stronger effect.

Statistics: Adequate, although the authors should indicate on the figures what regions of the gels were quantified.

Supplementary Results: The proteomic studies are not convincing because none of it is experimentally verified. Moreover, the nature of the changes in protein levels could have many explanations, none of them related directly to eff expression.

General comments:

The general approach to use Htt-polyQ to mimic the effect of E2 enzymes on protein aggregates is valid. To extrapolate that the turnover of Htt-polyQ by Eff is related to aging specifically, and not generally to cellular homeostasis is less convincing.

RNAi of certain E2's resulted in reduced amount of Htt aggregates, suggesting they contribute to the formation rather than degradation of these protein complexes. Is there a cumulative effective of knocking down multiple E2's that contribute to this phenomenon? Are deletion mutants available, and have they been examined for Htt aggregate formation?

The authors identified four E2's that promoted Htt degradation. Among these eff was examined further. Since RNAi of eff alone could reduce the turnover of Htt, it appears that its function is not fully overlapping with Ubc6, Ubc10, and Ubc84D. For instance, there may be multiple targets for ubiquitination in the path for Htt-polyQ degradation, including the aggregated protein itself, but also the proteasome, and other regulatory components. What is the effect of RNAi of multiple E2's simultaneously? Do the aggregates appear earlier, or are there higher amounts? If not, how does one explain their collective effect on Htt degradation?

The rescue of the Htt degradation defect caused by RNAi of either effort Ubc6 by human homologs is exciting and convincing.

One consideration that the authors should address is whether the turnover of other normally degraded proteins is reduced when eff (or Ubc6) expression is blocked by RNAi. Aggregated proteins, such as Htt-polyQ, may form unproductive interactions with the proteasome, and thereby interfere with the degradation of other cellular proteins. This indirect effect on proteasome function could cause aging-specific effects. In the fly model used in this work, is there any evidence that proteasomes co-localize with the Htt-GFP aggregates? It is important to establish that proteasome activity is unaffected, especially since ubiquitination of certain proteasome subunits can affect catalytic activity. This is a straightforward assay that can be measured using readily available fluorogenic substrates. Amore general effect of RNAi against eff is suggested by the increased levels of total polyUb proteins in both soluble and insoluble fractions of skeletal muscle cells.

If eff or Ubc6 regulate proteasome activity by ubiquitination, the results would be similar, but would not be due to a failure to ubiqutinate Htt-polyQ.

The authors should determine if the fluorescence generated by Htt-GFP is altered once it is present in an aggregate. If fluorescence is increased or decreased, it would affect the interpretation of their results. Reflecting on the work of Johnston and Kopito on the dynamic presence of CFTR-GFP aggregates in aggresomes, they observed that this process is reversible. It would therefore be useful to know if Htt-GFP can also be released from aggregates (and degraded) once eff expression is restored.

The authors do not show that Htt is the target of eff: only that there is a correlation between eff levels and Htt presence in aggregates.

Whereas the expression of human UBE2D2 rescued the retinal defect caused by RNAi shut down of eff expression, a similar rescue of insoluble aggregates in muscle tissue was not observed. Since the authors presume that eff reduces the levels of protein aggregates, shouldn't they have seen a decrease in insoluble aggregates with the expression of human UBE2D2? Does the prolongation of lifespan by expression of human UB2D2 indicate that only soluble aggregates are potentially toxic and result in aging?

Analysis of eff-induced protein changes revealed many house keeping proteins with altered expression (Fig. 5F). Whether they represent genuine targets of eff is unclear. Changes in these protein levels could simply reflect altered proteasome function arising from altered levels of aggregated Htt-polyQ or other aggregated proteins. These mass spec proteomic studies are

unconvincing and not informative because none of the proteomic findings is experimentally validated.

Based on the elevated levels of Pomp assembly factor the authors should investigate if proteasome assembly is altered. This simple test will address two questions. 1. Is proteasome activity altered, and 2, is proteasome composition altered? Simple experiment.

The observation by other investigators that UBE2D interacts with the CHIP E3 ligase in the turnover of misfolded proteins, and heat stress in yeast, is strongly consistent with the work described here.

The authors recognize the cellular targets other than Htt may be the prime contributors to aggregate-induced aging, they do not address the possibility of proteasome poisoning by accumulating levels of insoluble proteins. Therefore, it seems prudent to perform some studies to investigate proteasome assembly and catalytic activity, as well as polyubiquitination of Htt-polyQ in the presence and absence of eff. The proteomic studies are essentially phenomenological, and do not provide mechanistic insight because none of the hits was experimentally characterized.

The authors do not explain why they selected eff for further characterization, rather than Ubc6 which appeared to have a stronger effect on Htt-GFP accumulation (Fig. 1).

In Fig 3E the authors should indicate on the figure what regions of the gel/lane was quantified (3F). Same for Fig. 4.

In the lifespan studies (Fig 4D, E), why is the median lifespan 50 days versus 70 days for the controls?
