# Supplementary material for: The ubiquitin-conjugating enzyme UBE2D maintains a youthful proteome and ensures protein quality control during aging by sustaining proteasome activity
Source: PLoS Biol. 2025 Jan 29;23(1):e3002998. doi: 10.1371/journal.pbio.3002998 (PMC11778781; doi:10.1371/journal.pbio.3002998)
Supplement: Attachment — Submitted filename: eff_RESPONSE TO THE REVIEWERS.pdf [file pbio.3002998.s011.pdf]

**RESPONSE TO THE REVIEWERS' COMMENTS (PBIOLOGY-S-24-01712)**

Rev. 1:
Overall Recommendation: Minor Revisions. This is an interesting paper with good experimental data. Minor experimental and a few editorial additions will make it suitable for publication in PLOS Biology.
Novelty: This is an exciting line of investigation, and the hypothesis and experimental plan is novel.
Significance is Moderate. The significance of this work could be increased if it can be shown where eff E2 enzyme functions, and whether it has a general effect of overall aggregate turnover, or more specifically on proteasome function, or some other key step in protein turnover.
Technical merits: The experimental approach is sound, and the results are convincing. However, some improvements are recommended. Measurement of proteasome activity will determine if eff affects the function of this multicatalytic protease. Measurement of proteasome assembly will similarly test this. These experiments are important for completion of this solid body of work, and can be performed simultaneously in a native gel. The authors should explain why they selected eff for further investigation, rather than Ubc6, which seemed to have a stronger effect.
Statistics: Adequate, although the authors should indicate on the figures what regions of the gels were quantified.
Supplementary Results: The proteomic studies are not convincing because none of it is experimentally verified. Moreover, the nature of the changes in protein levels could have many explanations, none of them related directly to eff expression.

Response: Thank you for the insightful comments and the positive evaluation of our work. We have now amended the manuscript to address all the points raised and included new experimental results, which are presented in Fig. 5 and S1-S2 Fig. A detailed response to each specific point is provided here below.

General comments:
The general approach to use Htt-polyQ to mimic the effect of E2 enzymes on protein aggregates is valid. To extrapolate that the turnover of Htt-polyQ by Eff is related to aging specifically, and not generally to cellular homeostasis is less convincing.

Response: We have now further examined how eff silencing influences general cellular homeostasis by testing proteasomal and autophagic activity, two major general protein turnover pathways. Our new analyses are reported in Fig. 5 and indicate that UBE2D/eff is necessary to maintain optimal proteasome function: UBE2D/eff knockdown reduces the proteolytic activity of the proteasome, and this is rescued by transgenic expression of human UBE2D2 (homologous to eff). We also analyzed LC3/Atg8 levels and processing but found no major changes in response to eff knockdown (Fig. 5), suggesting that autophagy is not substantially affected by UBE2D/eff knockdown.

RNAi of certain E2s resulted in reduced amount of Htt aggregates, suggesting they contribute to the formation rather than degradation of these protein complexes. Is there a cumulative effect of knocking down multiple E2s that contribute to this phenomenon? Are deletion mutants available, and have they been examined for Htt aggregate formation?
The authors identified four E2s that promoted Htt degradation. Among these eff was examined further. Since RNAi of eff alone could reduce the turnover of Htt, it appears that its function is not fully overlapping with Ubc6, Ubc10, and Ubc84D. For instance, there may be multiple targets for ubiquitination in the path for Htt-polyQ degradation, including the aggregated protein itself, but also the proteasome, and other regulatory components. What is the effect of RNAi of multiple E2s simultaneously? Do the aggregates appear earlier, or are there higher amounts? If not, how does one explain their collective effect on Htt degradation?

Response: While it would be interesting to combine multiple RNAi lines targeting different E2s to test their collective impact on Htt levels, this experiment is not easily done because the combination of transgenes is

limited by the fact that most RNAi lines have been generated by integration into single genomic loci on chromosomes 2 and 3, which limits the availability of transgenes that could be combined for these studies. Moreover, combining multiple transgenes may reduce the efficacy of each transgene because of Gal4 titration effects (i.e., less Gal4 would be available to drive the expression of each UAS transgene). Nonetheless, we have now amended the manuscript to discuss the possibility that distinct E2s that affect Htt-polyQ-GFP aggregates may act via distinct or overlapping pathways, such as the modulation of proteolytic pathways (i.e. proteases, the autophagy/lysosome, and the ubiquitin/proteasome system) or by directly promoting the ubiquitination and degradation of Htt.

The rescue of the Htt degradation defect caused by RNAi of either effort Ubc6 by human homologs is exciting and convincing.

Response: Thank you.

One consideration that the authors should address is whether the turnover of other normally degraded proteins is reduced when eff (or Ubc6) expression is blocked by RNAi. Aggregated proteins, such as Htt-polyQ, may form unproductive interactions with the proteasome, and thereby interfere with the degradation of other cellular proteins. This indirect effect on proteasome function could cause aging-specific effects. In the fly model used in this work, is there any evidence that proteasomes co-localize with the Htt-GFP aggregates? It is important to establish that proteasome activity is unaffected, especially since ubiquitination of certain proteasome subunits can affect catalytic activity. This is a straightforward assay that can be measured using readily available fluorogenic substrates.

Response: Thank you for pointing out the possibility that proteasome activity could be impacted by UBE2D/eff knockdown. This is a valid hypothesis because, in addition to participating in target protein ubiquitination, a few E2 and E3 enzymes were previously found to associate with the proteasome: these proteasome-interacting proteins can modulate proteasome function and increase the resistance to homeostatic challenges. This hypothesis is particularly interesting in the case of UBE2D/eff because UBE2D/eff is homologous to the yeast E2 enzymes UBC4 and UBC5 that were previously reported to bind to the proteasome. Based on this premise, we have now measured whether UBE2D/eff knockdown modulates the proteolytic activity of the proteasome and found it to be the case. Specifically, we now report in Fig. 5 that UBE2D/eff knockdown reduces the proteolytic activity of the proteasome in skeletal muscle and that these defects can be rescued by expression of human UBE2D2 (i.e., one of its human homologs): this indicates that UBE2D/eff is necessary to maintain proteasomal activity (Fig. 5), which is key for proteostasis. The capacity of UBE2D/eff to modulate proteasome activity may therefore explain the role of UBE2D/eff in proteostasis during skeletal muscle aging (Fig. 3-7) as well as in the context of Huntington's disease (Fig. 1-2).

A more general effect of RNAi against eff is suggested by the increased levels of total polyUb proteins in both soluble and insoluble fractions of skeletal muscle cells. If eff or Ubc6 regulate proteasome activity by ubiquitination, the results would be similar, but would not be due to a failure to ubiquitinate Htt-polyQ.

Response: We agree with this interpretation and propose that UBE2D/eff maintains proteostasis in the context of Huntington's disease and skeletal muscle aging by sustaining the activity of the proteasome, as shown in Fig. 5. Concerning the alternative hypothesis that UBE2D/eff interacts with and ubiquitinates huntingtin, we have performed an IP-MS analysis of Htt in Drosophila but did not detect any interaction between UBE2D/eff and Htt, suggesting that Htt is not a ubiquitination target of eff/UBE2D. This new data is reported in the S5 Data file.

The authors should determine if the fluorescence generated by Htt-GFP is altered once it is present in an aggregate. If fluorescence is increased or decreased, it would affect the interpretation of their results. Reflecting on the work of Johnston and Kopito on the dynamic presence of CFTR-GFP aggregates in aggresomes, they

observed that this process is reversible. It would therefore be useful to know if Htt-GFP can also be released from aggregates (and degraded) once eff expression is restored.

Response: While additional mechanisms of UBE2D/eff-mediated proteostasis are possible (including a change in the biophysical properties of Htt-polyQ-GFP), our new data indicates a key role for UBE2D/eff in modulating proteasome activity (Fig. 5). Because of the key role of the proteasome in protein quality control, we propose that this is a key mechanism via which UBE2D/eff ensures proteostasis and cellular homeostasis.

The authors do not show that Htt is the target of eff: only that there is a correlation between eff levels and Htt presence in aggregates.

Response: We propose that Htt is modulated by UBE2D/eff via the capacity of UBE2D/eff to modulate the proteolytic activity of the proteasome (and not necessarily by direct ubiquitination of huntingtin-polyQ). We have amended the manuscript accordingly. To further address this question, we have also performed an IP-MS analysis of Htt in Drosophila but did not detect any interaction between UBE2D/eff and Htt, suggesting that Htt is not a ubiquitination target of eff/UBE2D. This new data is reported in the S5 Data file.

Whereas the expression of human UBE2D2 rescued the retinal defect caused by RNAi shut down of eff expression, a similar rescue of insoluble aggregates in muscle tissue was not observed. Since the authors presume that eff reduces the levels of protein aggregates, shouldn't they have seen a decrease in insoluble aggregates with the expression of human UBE2D2?

Response: Our new analyses reported in Fig. 5 indicate that eff impacts the proteolytic activity of the proteasome. On this basis, we propose a model whereby UBE2D/eff promotes the degradation of soluble Htt-polyQ by improving proteasome activity. In this model, larger aggregates (which cannot be readily degraded by the proteasome) accumulate in response to eff knockdown because of decreased proteasomal-mediated degradation of soluble Htt-polyQ. Soluble oligomers of aggregation-prone proteins such as Htt-polyQ are considered the most toxic species because of their capacity to bind to and interfere with the function of endogenous proteins.

Does the prolongation of lifespan by expression of human UB2D2 indicate that only soluble aggregates are potentially toxic and result in aging?

Response: The rescue of lifespan by expression of human UBE2D2 is only partial (i.e., the lifespan is not restored to control levels) presumably because of slightly different activities and target affinities of human UBE2D2 compared to Drosophila eff. In support of this model, $eff^{RNAi}$-modulated proteins such as Arc1, Arc2, Gnmt, and CG4594 are rescued by hUBE2D2 expression but $eff^{RNAi}$-induced changes in other proteins (e.g., UBL3) are not. Overall, these findings suggest that the hUBE2D2-mediated rescue of soluble ubiquitin levels contributes to lifespan but is insufficient for obtaining a complete rescue (insoluble ubiquitin would also need to be rescued to restore maximum lifespan).

Analysis of eff-induced protein changes revealed many house keeping proteins with altered expression (Fig. 5F). Whether they represent genuine targets of eff is unclear. Changes in these protein levels could simply reflect altered proteasome function arising from altered levels of aggregated Htt-polyQ or other aggregated proteins. These mass spec proteomic studies are unconvincing and not informative because none of the proteomic findings is experimentally validated.

Response: A major limitation for further validating eff targets obtained from TMT is the lack of antibodies to examine these proteins by western blots in Drosophila. However, we have now tested whether some of the proteomic changes induced by $eff^{RNAi}$ arise from a transcriptional stress response and found it to be the case. In particular, the upregulation of proteostasis regulators induced by $eff^{RNAi}$ (i.e., the chaperones Hsp22, Hsp26, Hsp70, and Hsp83 and the proteasome maturation factor Pomp) occurs also at the mRNA level. This new data is shown in S2 Fig. On this basis, we have modified the abstract, results, discussion, and summary scheme in

Fig. 8 to indicate that some of the protective proteins that are upregulated upon eff$^{RNAi}$ are induced transcriptionally, presumably as part of a stress response to the loss of proteostasis caused by eff knockdown.

Based on the elevated levels of Pomp assembly factor the authors should investigate if proteasome assembly is altered. This simple test will address two questions. 1. Is proteasome activity altered, and 2, is proteasome composition altered? Simple experiment.

Response: Assessment of the assembled proteasome and component stoichiometry often requires affinity purification of a tagged proteasomal component (https://pmc.ncbi.nlm.nih.gov/articles/PMC6427908/). We are unaware of any flies with tagged proteasomal components that have been thoroughly tested to perform these studies in Drosophila. However, the proteasome activity assays that we have performed (Fig. 5) indirectly answer this question because they provide a functional readout of the activity of assembled proteasome and how eff/UBE2D influences it.

The observation by other investigators that UBE2D interacts with the CHIP E3 ligase in the turnover of misfolded proteins, and heat stress in yeast, is strongly consistent with the work described here. The authors recognize the cellular targets other than Htt may be the prime contributors to aggregate-induced aging, they do not address the possibility of proteasome poisoning by accumulating levels of insoluble proteins. Therefore, it seems prudent to perform some studies to investigate proteasome assembly and catalytic activity, as well as polyubiquitination of Htt-polyQ in the presence and absence of eff. The proteomic studies are essentially phenomenological, and do not provide mechanistic insight because none of the hits was experimentally characterized.

Response: As explained above, we have now performed proteasome activity assays to address these questions and found that UBE2D/eff is necessary for optimal proteasome activity (Fig. 5). We have also examined the Htt-polyQ interactome but did not detect any interaction with eff (S5 Data), suggesting that Htt is not a direct ubiquitination target of eff, and that UBE2D/eff modulates Htt-polyQ aggregates because of its capacity to sustain the proteolytic activity of the proteasome.

The authors do not explain why they selected eff for further characterization, rather than Ubc6 which appeared to have a stronger effect on Htt GFP accumulation (Fig. 1).

Response: We selected UBE2D/eff for further analysis in muscle because eff is the E2 that is most downregulated during skeletal muscle aging in Drosophila (Fig. 3A). Moreover, we had already examined in a previous study (PMID: 33658508) the role of Ubc6 in muscle proteostasis and Htt-GFP.

In Fig 3E the authors should indicate on the figure what regions of the gel/lane was quantified (3F). Same for Fig. 4.

Response: This has been now indicated in Fig. 3-4. For the soluble fraction, the whole lane of poly-ubiquitinated proteins was quantified. For the insoluble fraction, the part of the lane above the major 40 KDa band was quantified.

In the lifespan studies (Fig 4D, E), why is the median lifespan 50 days versus 70 days for the controls?

Response: The controls in Fig. 4D and Fig. 4E have different median lifespans because of the differential effects of heterosis in different genetic crosses. Specifically, in Fig. 4D Mhc-Gal4 females were crossed with individual RNAi lines whereas in Fig. 4E Mhc-Gal4 females were crossed with combinations of overexpression lines and RNAi lines.

-------------------------
Rev. 2:
In this manuscript, Hunt et al. performed a comprehensive genetic study on ubiquitin-conjugating enzymes (E2s) using the Drosophila retina Htt-polyQ model. Based on the results of this targeted RNAi screen, they decided to focus on UBE2D/eff, which was among the few E2s that, upon knock-down, increased Htt-polyQ aggregation. They then tested the role of UBE2D/eff in skeletal muscle and provided compelling genetic, biochemical, and proteomics data indicating the age-associated decline of UBE2D/eff protein levels contributes to the loss of proteostasis in aging and lifespan shortening. Transgenic expression of human UBE2D2 partially rescued the lifespan deficit and proteomic remodeling resulting from eff RNAi, supporting an evolutionarily conserved role of UBE2D in maintaining proteostasis during aging. Overall, this study was carefully designed and performed, and the manuscript was well-written. Nevertheless, the authors should consider addressing the following comments to strengthen the paper further.

Response: Thank you for the positive evaluation of our work and for the excellent suggestions.

Major points:
1. The authors performed careful RNAi analysis by including multiple RNAi lines targeting a single corresponding gene (Fig. 1B&C). They conclude that the variability is likely due to the efficacy of different RNAis. This should be at least tested for eff by RT-qPCR (as in Fig. 4C), which will justify the authors' choices of RNAi in the following studies. In Fig.1B, among the 7-8 RNAis against eff, only 2-3 significantly increased Htt72Q-GFP. Do those lines have the best RNAi efficiency compared to the others? Are they used in the following studies in muscle?

Response: The two $eff^{RNAi}$ lines that increased Htt72Q-GFP in the screen (Fig. 1 and S1 Data) are BL35431 (which induced the highest increase in Htt72Q-GFP levels) and BL31875. We now show in Fig. S2 that these two RNAi lines have different efficacy in knocking down eff. Specifically, BL35431 (eff RNAi line #2) induces a >75% eff knockdown, compared to a ~40% eff knockdown obtained with BL31875 (eff RNAi line #1). On this basis, the strongest $eff^{RNAi}$ line (BL35431) was utilized for experiments in Fig. 2-7. The methods have now been updated to mention that BL35431 is the $eff^{RNAi}$ line that was preferentially used because of its higher efficacy.

2. The p62 levels increase upon eff RNAi in the detergent-soluble fraction (Fig.3E&F, Fig.4 A&B), and likely in the detergent-insoluble fraction as well based on Fig. 4A&B. Do the data suggest an impairment of autophagy? The author concluded that the result supports the role of eff in protein quality control without providing a detailed explanation.

Response: One of the functions of p62 consists of its capacity to work as an autophagic adaptor protein that binds to insoluble poly-ubiquitinated proteins and promotes their removal via autophagy. An increase in the insoluble levels of p62 can, therefore, indicate a defect in the autophagic removal of the poly-ubiquitin proteins to which p62 binds. To test whether autophagy is modulated by eff knockdown, we have monitored LC3/Atg8 but found no substantial changes in its total levels and processing (detergent-soluble Atg8-II/I levels). However, there was a significant decrease in the detergent-insoluble levels of Atg8-II/I, which may indicate subtle defects in autophagy. This new data is shown in Fig. 5D-G. Together with the new analysis of proteasome activity (Fig. 5A-C), these studies collectively indicate that insoluble poly-ubiquitinated proteins accumulate upon eff knockdown because of decreased proteasome activity: p62 binds to these insoluble poly-ubiquitinated proteins but there is no substantial autophagy induction to remove them.

3. A difference between the eff-RNAi-induced proteins in Fig. 5F and 5G is: proteins in Fig. 5F decreased upon transgenic expression of UBE2D2 (without eff RNAi), while proteins in Fig.5G did not change. This supports the authors' idea that proteins in 5G are a protective response. The author could also test if the induction of proteins in 5G occurred at the mRNA levels (esp. the chaperones as part of the heat shock

response).

Response: We have tested whether the expression of chaperones (Hsp22, Hsp26, Hsp70, and Hsp83) and Pomp (which is induced by eff$^{RNAi}$, as shown in previous Fig. 5F-G, now Fig. 6F-G) are modulated at the mRNA levels and found it to be the case. This new data is shown in S2 Fig. On this basis, we have modified the abstract, results, discussion, and summary scheme in Fig. 8 to indicate that some of the protective proteins that are upregulated upon eff$^{RNAi}$ are induced transcriptionally, presumably as part of a stress response to the loss of proteostasis caused by eff knockdown.

Minor points:

1. The authors used p62/Ref(2)P and Ref(2)P/p62 at different places. Similarly, they used both UBE2D/eff and eff/UBE2D. It is better to keep the nomenclature consistent throughout the manuscript.

Response: This has been corrected throughout the manuscript: p62/Ref(2)P and UBE2D/eff have been used consistently.

2. The labeling of sample names in Fig.4A (eg. "control + mCherry") is very busy.

Response: A color guide has been added above the sample names in Fig. 4A to improve the legibility of this figure.

3. In Fig. 5F, although those eff target genes likely increase protein levels upon eff RNAi due to "improper eff/UBE2D-mediated turnover", the authors did not formally rule out the possibility that the changes could occur at protein synthesis. They may want to soften the statement.

Response: Thank you for pointing this out. We have modified this sentence and removed "UBE2D-mediated turnover". The revised sentence reads as follows: "Altogether, these proteomics analyses identify proteins that are modulated by UBE2D/eff in skeletal muscle (Fig. 6F) and that may contribute to deranging muscle protein quality control and reduce survival."

4. In the discussion, the authors proposed that upon eff RNAi, misfolded proteins may clog or burden the proteasome, which is a very reasonable idea. Given the role of eff in mitophagy, eff RNAi may also interfere with the autophagy-lysosome system contributing to the loss of proteostasis.

Response: This is a very good point. We have monitored autophagy with anti-LC3/Atg8 antibodies but found no substantial changes in the total levels of Atg8 and Atg8 processing (detergent-soluble Atg8-II/I levels). However, there was a significant decrease in the detergent-insoluble levels of Atg8-II/I, which may indicate subtle defects in autophagy. This new data is shown in Fig. 5D-G.
